# Collagens of Poriferan Origin

**DOI:** 10.3390/md16030079

**Published:** 2018-03-03

**Authors:** Hermann Ehrlich, Marcin Wysokowski, Sonia Żółtowska-Aksamitowska, Iaroslav Petrenko, Teofil Jesionowski

**Affiliations:** 1 Institute of Experimental Physics, TU Bergakademie Freiberg, Leipziger str. 23, 09599 Freiberg, Germany; iaroslavpetrenko@gmail.com; 2 Institute of Chemical Technology and Engineering, Faculty of Chemical Technology, Poznan University of Technology, Berdychowo 4, 61131 Poznan, Poland; marcin.wysokowski@put.poznan.pl (M.W.); sonia.zoltowska-aksamitowska@doctorate.put.poznan.pl (S.Ż.-A.); teofil.jesionowski@put.poznan.pl (T.J.)

**Keywords:** collagen, spongin, collagen-related proteins, sponges, scaffolds, biomaterials

## Abstract

The biosynthesis, structural diversity, and functionality of collagens of sponge origin are still paradigms and causes of scientific controversy. This review has the ambitious goal of providing thorough and comprehensive coverage of poriferan collagens as a multifaceted topic with intriguing hypotheses and numerous challenging open questions. The structural diversity, chemistry, and biochemistry of collagens in sponges are analyzed and discussed here. Special attention is paid to spongins, collagen IV-related proteins, fibrillar collagens from demosponges, and collagens from glass sponge skeletal structures. The review also focuses on prospects and trends in applications of sponge collagens for technology, materials science and biomedicine.

## 1. Introduction

Collagens constitute a superfamily of long-lived extracellular matrix structural proteins of fundamental evolutionary significance, found in both invertebrate and vertebrate taxa. They are among the most studied proteins due to their important functions in mammals, including humans. In addition to their structural function in cartilage and skin formation [1,2], as well as in the biomineralization of hard tissues [3] including bone [4] and dentine [5], collagens are involved in the regulation of diverse cellular functions and processes. During the last 60 years, research into collagens has evolved from the discovery of the structure of collagen [6,7], through studies on its chemistry and biochemistry [8,9,10], to present-day applications in cell therapy [11], biomedicine [12,13,14], cosmetics [15], and the food industry [16]. A rod-like triple-helical domain is the typical structural element in all collagens. However, they differ in their size, dislocations of the globular domains and imperfections within the triple helix, self-assembly behavior, and functional roles. The classification of collagens is based on structural and functional features of vertebrate collagens. For example, 28 collagen types have so far been identified and characterized at the molecular level in mammals (see for review [1,17]). Collagens are also divided into subfamilies based on their supramolecular assemblies: fibrils, beaded filaments, anchoring fibrils, and networks [11]. Usually, the amino acid sequences in collagens are responsible for the corresponding functional properties: energy storage capacity, stiffness, or elasticity [18]. Even the type of amino acid motif within the tropocollagen molecule of a collagen can significantly affect its mechanical properties. Consequently, it can be hypothesized that the diversity of collagen polyforms determines their future functions, even within the same organism.

Marine vertebrate collagens have attracted scientific attention, mostly as products of fisheries [19]. In particular, fish-sourced collagens from skins and scales [20,21,22] have been studied and used as alternative collagen sources to avoid the potential risks associated with mammalian collagen due to bovine spongiform encephalopathy and the swine influenza crisis [23].

In contrast to marine vertebrate collagens, similar structural proteins found in marine invertebrates represent one of the most ancient protein families within Metazoa. Marine invertebrate collagens arose earlier than their vertebrate analogs, and possess diverse unique structural features, including very special structure–function interrelations. Collagens from poriferans, coelenterates, annelids, mollusks, echinoderms, and crustaceans have been discussed in detail in several review papers (e.g., [24,25,26,27,28,29,30,31,32]) and books (e.g., [33,34]). The limiting factors that have hindered progress in this field of research are the difficulty of purifying marine invertebrate collagens and their relative species-dependent complexity. However, there are more than enough examples in practically every order of marine invertebrates to inspire experts in materials science and biomedicine, especially because the similarities in structure and biosynthesis between vertebrate and invertebrate collagens appear to be more impressive than the differences [24].

Sponges (Porifera) are the most simple and ancient multicellular organisms on our planet, and mostly live attached to a suitable substratum (rock, sandy sediments) on the seabed. Poriferans diverged from other Metazoans earlier in evolutionary history than any other known animal phylum, extant or extinct [35], with the first fossilized sponge remnants found in 1.8 billion-year-old sediments [36,37,38,39,40,41]. The phylum Porifera is divided into four classes: Hexactinellida, Demospongiae, and Homoscleromorpha, with silica-based skeletons; and Calcarea, with a skeletal network made of calcium carbonates [42]. According to Exposito et al. [27], before the divergence of the sponge and eumetazoan lineages took place, the genes which were responsible for the synthesis of some kind of ancestral fibrillar collagen arose at the dawn of the Metazoa. The duplication events leading to the formation of the A, B, and C clades of the fibrillar collagens occurred before the eumetazoan radiation. Interestingly, the similarity in the modular structure of sponges and humans is preserved only in the B clade of fibrillar collagens. This phenomenon correlates well with the hypothesis of the primordial function of type V/XI fibrillar collagens in initiating the formation of collagen fibrils [27].

Different systems of terminology relating to poriferan collagens are found in the literature, as sponges also display considerable polymorphism with respect to their collagenous structures. The insolubility of most poriferan collagens has been the main obstacle to carrying out any detailed biochemical analysis. Studies on the morphology and nanotopography of the collagenous fibrils have shown that they are dispersed throughout the intracellular matrix within the skeletons of sponges. Cuticular structures have been found in some sponges, but their molecular composition has not been determined [43].

It was accepted very early that collagen fibers in sponges can possess quite different morphological features [44]. Gross et al. isolated two distinct forms of collagen from *Spongia graminea*, which they called spongin A and spongin B [29]. The first corresponds to fine intercellular collagen fibrils, visible only by electron microscopy. The second, spongin B, forms macroscopically-visible rigid fibers which are characteristic of keratosan demosponges [43]. This was probably the moment when the terminological divergence arose with regard to the term *spongin*, which was initially proposed by Städeler [45] to denote the skeletal fibrous matter of bath sponges, and was also used for spongins A and B defined by Gross et al. [29]. Up to the present, the authors of numerous publications—especially those on applications of spongin-based scaffolds in tissue engineering [31,46,47,48,49,50,51]—have used the term collagen for spongin, or even defined spongin as “collagenic skeleton” [52]. Very recently, Tziveleka et al. [53] studied collagen from the marine demosponges *Axinella cannabina* and *Suberites carnosus*, and proposed three different terms: insoluble collagen (InSC), intercellular collagen (ICC), and spongin-like collagen (SlC). It is worth noting that the isolation of each form of collagen from demosponges is based on the selectivity of the method used. Data on collagen extraction methods from diverse mineralized sponges (Hexactinellida, Demospongiae) and sponges which lack mineralized skeletons (the subclass Keratosa)—including yields of the extracted collagens—may be found in the relevant papers.

**Figure 1:**
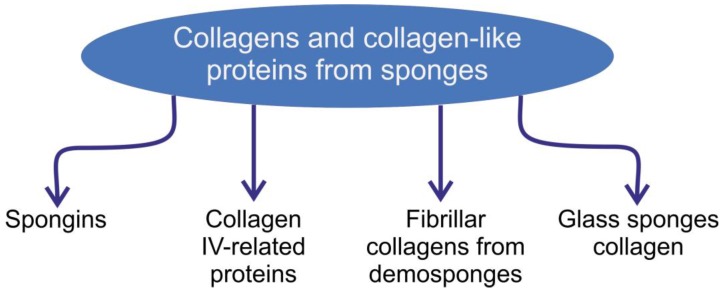
Schematic overview of the collagens and collagen-like structural proteins of poriferan origin described in this review.

In this review, we focus on the structural diversity of collagens and collagen-like proteins in selected sponges, with particular focus on their origin, structural features, and applications in biomedicine and technology, including materials science and biomimetics. The review has the ambitious goal of providing thorough and comprehensive coverage of poriferan collagens (Figure 1) as a multifaceted topic with controversial hypotheses and numerous open questions. We begin with a brief description of spongins and their practical applications. Next, we examine the collagen IV-related proteins in diverse representatives of Porifera. Special attention is paid to *Chondrosia* sp. collagens and their applications in marine biotechnology, biomedicine, and cosmetics. Finally, we discuss the current state of work related to the unique hydroxylated collagen discovered in anchoring siliceous spicules of psychrophilic deep-sea glass sponges. We are optimistic that both the attempts to establish implications for poriferan collagens and the numerous open questions raised in this review will inspire the scientific community to carry out research into collagens and collagen-related proteins from sponges, as ancient and intriguing structural biopolymers.

## 2. Spongins as Enigmatic Structural Proteins in Sponges

**Figure 2:**
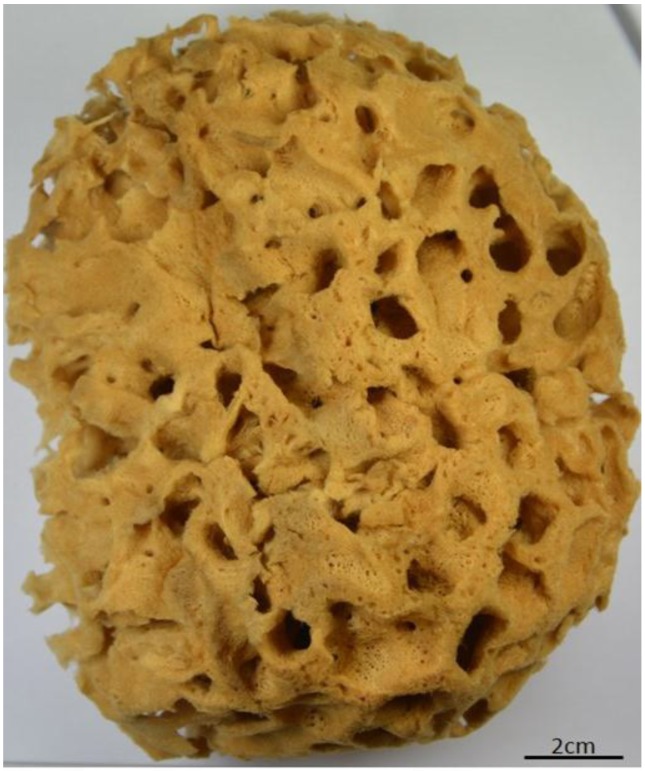
The mineral- and cell-free skeleton of commercial *Hippospongia communis* bath sponge is an example of a 3D spongin scaffold.

**Figure 3:**
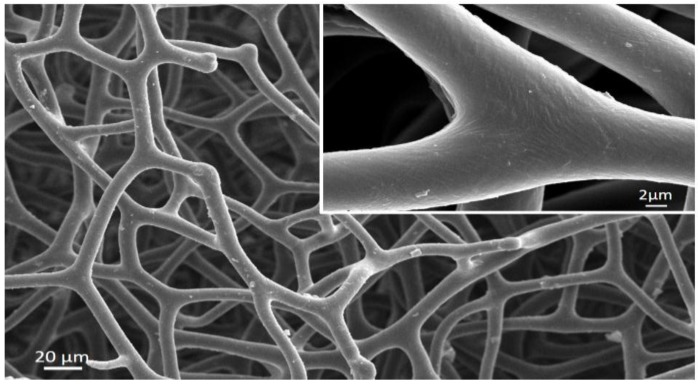
Scaning electron microscopy (SEM) image of anastomosed spongin fibers from the demosponge *H. communis*, which are organized as sets of unconnected structures with dendritic architecture.

It is recognized that so-called spongioblasts—derived from the epithelium of sponges—are responsible for the formation of spongin. Minchin claims that the fibers of skeletal spongin are formed extracellularly; however, the cuticular spongin fibrils are of intracellular origin [19]. In contrast to such structural proteins as collagen, fibroin (silk), elastin, resilin, and keratin, the chemistry and molecular biology—including the sequences—of spongins so far remain unknown. It seems that spongin is the last enigmatic proteinaceous biopolymer, although it is of very ancient origin and has undergone more than 300 years of investigations. Spongin in the form of cell-free skeletons of diverse bath sponges (Figure 2 and Figure 3) has been used for more than 3000 years [54,55] for painting, bathing, and cleaning, as padding for battle armor, for medical purposes, and as a vessel for drinking water [56]. A brief overview of the practical applications of spongin from bath sponges in biomedicine and technology in recent times is given in the next section.

A suggestion of a similarity between silk and bath sponge skeletal fibers was reported for the first time by Geoffroy in 1705 [57], and was based on his chemical experiments. After that, attention was paid to practical applications of sponges in pharmacology due to the presence of iodine in their skeletons. For example, in 1819, Andrew Fyfe—a professor of chemistry in Aberdeen—identified large quantities of iodine in the marine sponge *Spongia usta*, the “Coventry Remedy”, which was used even in ancient China [58]. In 1841, bath sponges were described as those in which the essential base of the skeleton consists of keratose fibrous matter. At that time, the structural and chemical similarity between horny fibers of sponges and silk was again suggested by Croockewit [59]. It would appear that the horny matter of sponge is closely analogous to silk and related proteins, differing from them only in that it contains additional halogens. According to Croockewit, the chemical formula of horny matter must be as follows: 20(C_39_H_62_N_12_O_17_) + I_2_S_3_P_10_ [59]. Schlossberger [60], however, reported the very slight solubility of the fibrous matter in ammoniacal solution of copper hydroxide. Additionally, treatment with diluted sulfuric acid leads to the identification of leucine and glycocoll, in contrast to the isolation of tyrosine and serine from sericin under similar conditions. Städeler in 1859 obtained similar results [45] and introduced for the first time the scientific term *spongin* for this horny matter. Then, in 1864, von Kölliker [61] carried out the first histological studies on sponges, including investigations of the structural features of fibrous spongin. Diverse iodine-containing sponges and the matter termed as “*Jodospongin*” were discussed by Hundeshagen in 1895 [62]. The organic origin of iodine in bath sponge skeleton was suggested by Harnack [63]. He estimated the concentration of iodine in spongin at 1.1–1.2%, and demonstrated that superheated steam destroys the organic portion of spongin fibers completely, liberating iodine.

In 1898, Harnack isolated the “*Jodspongin*” and characterized it as an albuminoid-like product, containing over 8.5% iodine and 9.4% nitrogen [63]. In 1926, Clancey carried out a critical analysis of the literature to-date relating to the identification of spongin by other authors. In contrast to other physiologists, he suggested that the origin of the skeletal spongin in Euceratosa was not the same as that of the spongin which surrounds the spicules in the Pseudoceratosa [64]. At that time, the common bath sponge *Hippospongia equina* and the “Turkey cup sponge” *Euspongia officinalis* were the sponges most studied with respect to spongin. The results published in various papers [65,66] showed remarkable differences, due to insufficiently effective analytical methods and the use of commercial sponges that had been variously prepared and bleached. Consequently, different results on the chemical nature of spongins from particular species were obtained.

For example, Clancey [64] isolated up to 7% of iodogorgonic acid besides the other amino acids in acidic hydrolysates of spongin. Clancey [64] did not identify hydroxyproline in spongin fibers of *Hippospongia equina* which had been treated with acid and alkali. It should be noted here that in natural collagen, a 3(S)-hydroxy-l-proline (3-Hyp) residue occurs together with a 4-Hyp residue, which is known to markedly increase the conformational stability of the collagen triple helix [67]. Hydroxyproline is found almost exclusively in collagen [8]. Thus, Clancey found a remarkably high amount of glutamic acid (18.4%), as well as 14% glycine, 5.7% proline, 2.8% tyrosine, 11% tryptophan or histidine, and a trace of cystine. Block and Bolling [68] presented the following results on the chemistry of spongin (Table 1).

The content of glycine (about 14%) in this spongin is significantly lower than in collagen (between 25% and 33%) [8]. Thus, until the identification of two different spongins by Gross et al. in 1956, spongin was recognized, for the most part, as a halogenated scleroprotein (see Table 2) [69,70,71] or neurokeratin-like protein [68] due to the presence of cystine.

Consequently, it is very curious that the two morphologically-distinct forms of spongin fibers—termed spongin A and spongin B—were classified by Gross et al. [29] as members of the collagen family. This was probably because such an analysis was supported by electron microscopy and X-ray diffraction, and by their general amino acid pattern, including corresponding glycine and hydroxyproline content. Ratios of glycine to hydroxyproline were 1.6 and 1.8 for spongins A and B, respectively. The results obtained with small-angle X-ray diffraction and electron microscopy showed the diameter of the spongin A unbranched fibril to be on the order of 20 nm, with an axial period of about 650 Å. The large and branched fibers of spongin B were 10–50 μm in width and composed primarily of bundles of thin unbranched filaments less than 10 nm wide [29]. Both fiber types and the amorphous matrix contain hexosamine, hexose, pentose, and uronic acid. Glucosamine, galactosamine, glucose, galactose, mannose, fucose, arabinose, and uronic acid were identified chromatographically in both spongin A and in the amorphous substance. It was shown that spongin B contains a small amount of amino sugar plus glucose and galactose. In contrast to mammalian collagen, neither spongin can be dissolved at all by collagenase (*Clostridium hystolyticum*) or pepsin, nor were they dissolved to any appreciable extent in alkali solutions or dilute acid [29]. In a paper by Katzmann et al. [72], it was reported that spongin B accounts for over 70% of the dry weight of the bath sponge *H. gossypina*, and contains approximately 7% by weight of glucosylgalactosylhydroxylysine but a negligible amount of other sugars.

Recently, Langasco et al. [52] isolated glycosaminoglycans (GAGs) from sponginous skeletons of selected bath sponges. Total GAG content—expressed as μg hexuronate/mg dry weight—shows some variability among the tested species, being 0.171 ± 0.021, 0.367 ± 0.028, and 0.460 ± 0.081 for *H. communis*, *Spongia officinalis*, and *S. lamella*, respectively. The data obtained suggest that these sponge GAGs are structurally divergent from vertebrate GAGs [52].

Thus, it seems that spongin chemistry is made very complex by the presence of diverse halogens (I, Br) which have never been reported in natural collagens or keratins. This may explain the very high resistance of this proteinaceous biopolymer to enzymatic treatment. Its unique resistance to various enzymes—including amylases, lysozymes, trypsin, pronase, collagenases, and other proteases—is well reported [44,72]. On the other hand, in the natural environment diverse bacteria are able to destroy spongin enzymatically and lead to extremely high levels of damage to the structure of the spongin-based skeletal fibers (see for details [73]). The isolation and purification of such special “*sponginases*” remain a challenge for future research, and will provide a key way to obtain peptides that will be useful for detailed proteomic analysis and the sequencing of spongin.

**Figure 4:**
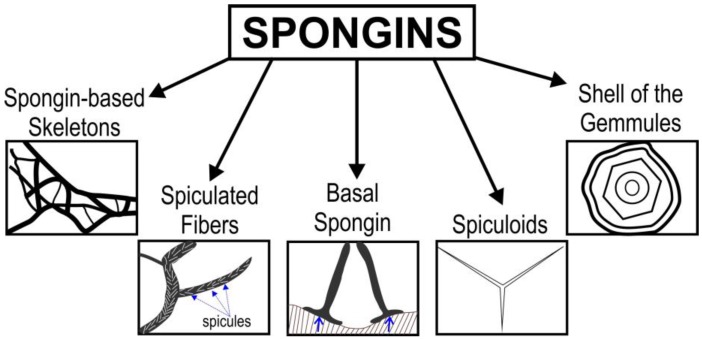
Diversity of spongins according to [43].

Understanding of the nature and origin of spongins—especially in keratosan demosponges (the orders Verongiida, Dictioceratida, and Dendroceratida)—changed dramatically after the discovery of chitin as a second structural component of the skeletal fibers of demosponges in the order Verongiida by the Ehrlich Group in 2007 [74,75,76]. It was shown that anastomosing and macroporous skeletons of diverse verongiids are made of some kind of spongin–chitin biocomposites. The content of chitin in such composites ranges between 10% and 60% depending on the sponge species [76]. The isolation and characterization of chitin in these composites was possible due to the well-known resistance of chitin to dissolution in alkaline solutions [77,78,79], in contrast to spongins, which are quickly dissoluble in alkali [72,80]. Consequently, all publications prior to 2007 on spongins found in Verongiida sponges must be re-examined. The only existing and up-to-date classification of spongins is that proposed by Garrone in 1978 [43]. He states that the following types of spongins can be defined and discussed (see Figure 4). The first spongin is to be found in the form of spiculated fibers. These structures are associated with the endogenous mineralized skeleton of the sponge. It is also responsible for the formation of wide fibers which include only a very thin mineral element in the core. This kind of spongin is also resistant to mild acid or alkaline hydrolysis, as well as to pepsin and diverse bacterial collagenases. However, this spongin can be partially destroyed by cuprammonium hydroxide treatment at room temperature.

Second are the spongin fibers which form the skeleton of the keratosan demosponges: the abundance and compactness of the spongin and the almost complete lack of its own inclusions—which are replaced with foreign particles—testify to the originality of the spongin in this group. A typical example of such spongin can be found in the genus *Ircinia*, characterized by spongin fibers cored with foreign debris (sand microparticles) [81]. Recently, Castritsi-Catharios et al. [82,83,84] described the chemical elements and the physical properties of such skeletal spongin from diverse commercial sponges before and after chemical treatment.

The importance of the so-called basal spongin is evident for all sponges as sessile animals. In sponges with no organized internal skeleton, the organism is attached to the substratum by a more or less continuous layer of external spongin. This spongin is secreted by the basopinacocytes. The basal spongin is continuous with the internal spongin only in poriferans with an organized skeleton, formed either of spongin fibers or spiculated fibers. Due to the function of the basal spongin in such demosponges as *Chondrosia reniformis* (a species lacking spicules and internal spongin), the animal is attached strongly to its substratum. The basal spongin is discontinuous in erect sponges, where it forms the starting points of the internal organized skeleton. However, in the endemic fresh water demosponge *Lubomirskia baicalensis*, the holdfast which is responsible for attaching the sponge body to the hard substratum contains both basal spongin and chitin [77].

The extremely flexible and elastic organic structures which are morphologically similar to mineralized spicules are known as spiculoids [85]. They have been described in representatives of the genera *Darwinella* and *Igernella* (order Dendroceratida), where they are either free or partly joined to the fibers of the skeleton. They are compressible and can be easily torn apart. Finally, spongin may be responsible for the protection of gemmule shells. Gemmules are formed within the tissues of most freshwater and some marine sponges, and represent morphologically diverse asexual reproductive spherical bodies a few tenths of a millimeter to more than 1 mm in diameter, composed of a dense mass of identical cells and surrounded by an organic coat called the shell. The shell of gemmules is fortified with siliceous spicules and gemmoscleres, which are embedded into a matrix composed of both chitin and a collagenous protein. This collagen has been referred to as spongin [43].

### Trends in the Applications of Spongins

The history of studies on the chemistry, molecular biology, biochemistry, and bioinspired materials science of spongins remains relevant today, partly due to the poorly understood basis of ecological disaster in the case of sponge diseases, but mostly due to recent progress in the direct applications of sponge skeletons as 3D spongin scaffolds in tissue engineering and biomimetics. Additionally, the marine ranching of bath sponges worldwide is a crucial factor in the adoption of spongins as renewable naturally prestructured proteinaceous scaffolds.

**Figure 5:**
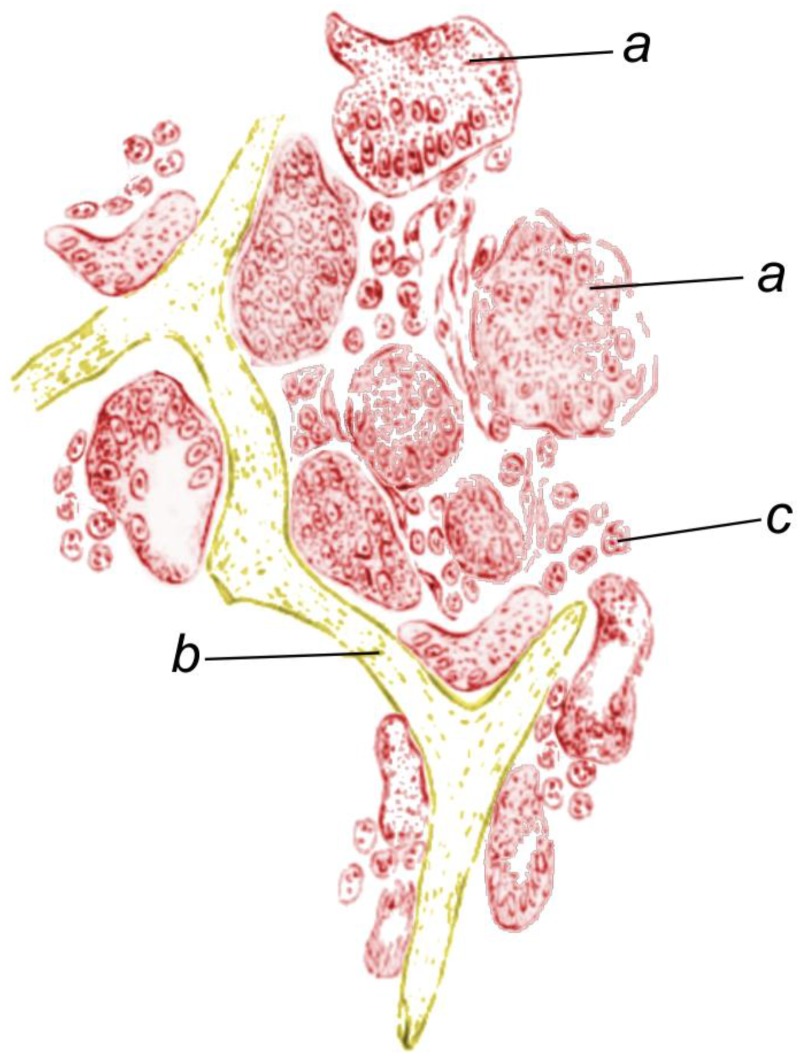
Sketch of a fragment of spongin framework (**b**) surrounded by a great number of living cells (**a**,**c**) in a sponge-grafting application (adapted from [92]).

The spongin-based skeletons of bath sponges appear to possess a number of unique and useful properties, which had been exploited long before such scientific fields as tissue engineering and bioengineering were proposed. As reviewed by Szatkowski et al. [86], from the 18th century commercial bath sponges were valued in medicine due to their softness, high compressive strength, ability to retain shape, and high sorption rates. For these reasons, they were used as compression bandages for pressing open sinuses, in overcoming strictures of body passages (including the rectum), for dilation of the cervix uteri [86,87,88,89,90], and in the form of sponge tents applied in the uterus to expand the cavity and enable examination. More intriguingly, fragments of sponge skeleton were used as small prostheses in early “plastic surgery” [91]. Revolutionary results were obtained by Hamilton in 1881. In a paper entitled “*On sponge-grafting*” [92], he reported the following case. A woman underwent surgery for removal of a mammary tumor, during which a large area of skin was removed. The skin was replaced with a thin slice of an aseptic sponge skeleton, which ten days after the surgery was observed to be vascular, and three months later was covered with epithelial tissue (Figure 5).

Today, spongin-based scaffolds are actively used in diverse applications related to tissue engineering. Positive results have been reported with human osteoprogenitor cells on the skeleton of *S. officinalis* [46], with osteoblast-like MG-63 cells growing on spongin from *Hymeniacidon sinapium* [93] and with mouse primarily osteoblasts on spongin from *Callyspongiidae* marine demosponges [49]. Recently, Nandi et al. [51] have proposed that the skeleton of the marine sponge *Biemna* sp.—alone and in combination with growth factors—is a promising biomaterial for bone repair and bone augmentation.

Besides applications in the biomedical field, spongin-based scaffolds have been successfully used as adsorbents of diverse dyes [94,95] and as supports for enzyme immobilization [96]. It was recently shown that spongins are thermostable up to 260 °C [86,97,98]. This property opens the door for applications of spongin-based scaffolds with 3D architecture in such novel scientific disciplines as Extreme Biomimetics [99], with the aim of developing novel advanced composite materials.

## 3. Collagen IV and Related Proteins in Sponges

It is now well established that collagens are key to the structural integrity and biomechanical properties of various tissues of Metazoans. One of them, the basement membrane-forming collagen IV, is extremely ancient. Collagen IV networks have a polygonal architecture that endows basement membranes (BMs) with a tensile strength sufficient to protect tissues from mechanical stress, in addition to serving as important regulators of the dynamic events associated with cell adhesion, signaling, and survival [100]. According to the modern view [101], only the presence of the collagen IV gene was precisely correlated with the emergence of BMs in animals. Thus, the triple helical collagen IV was required for the development of BMs.

BMs underlie the epithelia in Metazoa from sponges to humans [102]. Interestingly, until 1996, basement membrane structures and type IV collagen were known to be present in all multicellular animal species except sponges. In Porifera, BMs are associated with the basal surfaces of polarized epithelial cells [103]. After the first report on the identification of type IV collagenous sequences in the homoscleromorph sponge *Pseudocorticium jarrei* by cDNA and genomic DNA [103], this collagen has been found in diverse poriferans. For example, in corresponding transcriptome data from a calcareous sponge (*Sycon coactum*) and another homoscleromorph sponge (*Corticium candelabrum*), two new type IV collagen genes were found in each [104]. Homologs of important components of basement membrane genes, including type IV collagen, have been found in the Demospongiae *Spongilla lacustris*, *Ircinia fasciculata*, and *Chondrilla nucula* [105]. The discovery of type IV collagen in Calcarea and Demospongiae is very important, because nowhere in this group has a BM-like structure been noted. The presence of type IV collagen in glass sponges (Hexactinellida) remains to be detected. Polyclonal antibodies have detected type I (but not type IV) collagen in the anchoring spicules of the *Hyalonema sieboldii* glass sponge [3].

The relationship between type IV collagen and the so-called spongin short chain collagen (SSCC) [106] is still under investigation [101]. SSCC has been considered as ancestral to type IV collagen [107]. Like type IV collagen, SSCC also has NC1 domains which produce the globular heads particular to type IV collagen and which are required for assembly of the unique scaffold of the BM (see for review [104]). It is suggested that collagen IV and its spongin variant are primordial components of the extracellular microenvironment, where collagen IV especially was a key player in the evolution of epithelial tissues in Metazoa, including sponges, due to the transition to multicellularity [101].

Interestingly, collagen IV from the demosponge *Chondrosia reniformis* has recently been patented as a source of special membranes for biomedical applications [108]. The collagen was isolated with an extraction solution of 100 mM Tris-HCl, 10mM EDTA, 8 M urea, and 100 mM 2-mercaptoethanol, rendering the protein in the form of a precipitate. This was used for the development of stable and non-cytotoxic type IV collagen membranes, which can be applied in tissue engineering and regenerative medicine approaches for epithelial repair, regeneration, or replacement. The technology includes the re-epithelialization of any single and stratified epithelium, with emphasis on the skin.

## 4. Fibrillar Collagens in the Mesohyl of Demosponges

The mesohyl includes a noncellular colloidal mesoglea with embedded collagen fibers, spicules, and various cells, being as such a type of mesenchyme. It is currently debated whether the mesohyl and pinacoderm layers in sponges are true tissues [109]. Collagens serve several functions in sponges [27,106,110]. The formation of mesohyl certainly involves the activity of fine fibrils made of fibrillar collagen. The collagen fibrils both mediate cell–matrix interactions via membrane receptors and provide the structure of the extracellular matrix (ECM), a situation observed in vertebrates. The increase in the structural diversity of fibrillar collagen chains, their different forms of maturation, and interactions with other ECM components appeared during the process of evolution [111]. The diversity of sponges which contain high amounts of fibrillar collagen within their mesohyl has been described previously (see for review [110,111,112]).

Fibrillar bundles, formed by the association of several hundred collagen fibrils, have been observed in diverse species of *Tethya*, *Chondrosia*, *Chondrilla*, *Jaspis*, and *Suberites* (see for details [112,113]). The densely packed bundles of collagen fibrils are secreted exclusively by the highly polarized lophocyte cells [43,111]. These are actively moving cells, pulling behind them a bundle of regularly arranged collagen fibrils.

**Figure 6:**
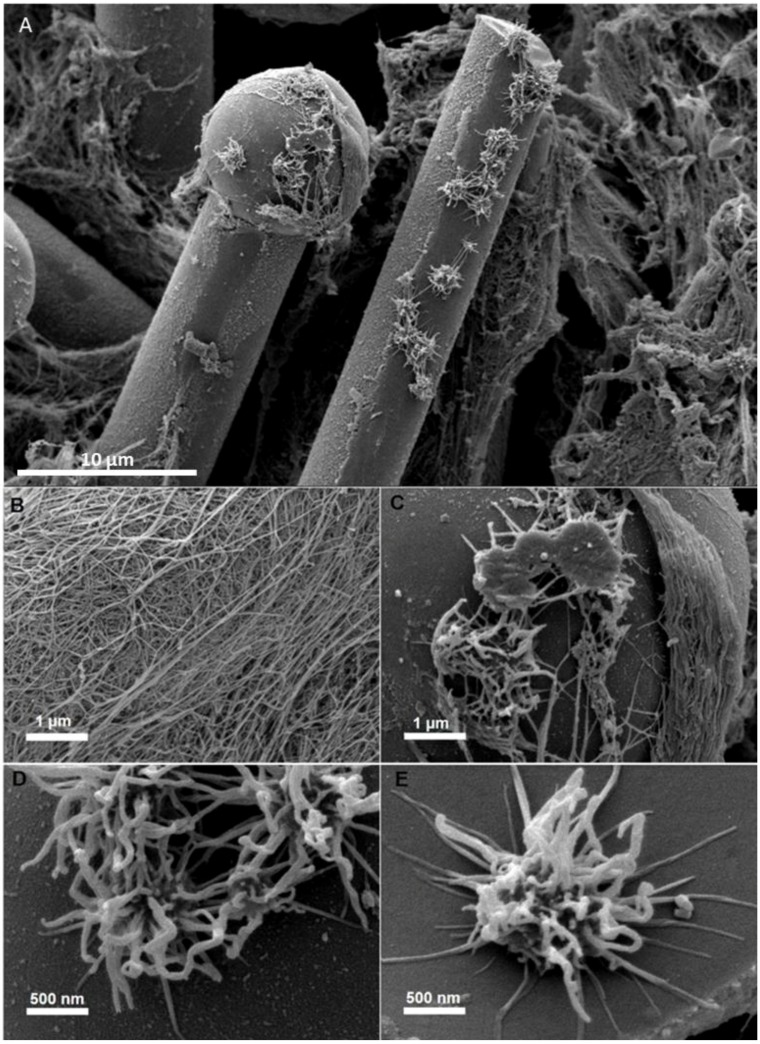
SEM view through the collagenous mesohyl of the demosponge *S. domuncula*. Layers of collagen fibrils (**A,B**) are a result of the activity of the unique collagen-producing cells which are seen to line up along the surface of the spicules (**C**–**E**). The line of cells (**A**) can move from left to right along the spicule, depositing a rough, nanofibrillar collagenous layer in their wake (**C**) (see also [114]).

Another kind of collagen-producing cell has been discovered in the mesohyl of the demosponge *Suberites domuncula* [114,115], in which the expression of collagen genes is controlled by silicate and myotrophin [116]. SEM observations have revealed the complex collagen network surrounding the spicules within the mesohyl of adult specimens (Figure 6).

Collagen fibers have also been identified in the mesohyl of the demosponge *Haliclona rosea* [116]. Collagen has also been reported in the mesohyl of such Calcarea sponges as *Leucosolenia* sp. and *Leucandra* sp. [117].

Collagen fibril content is also high in the external asexual buds that occur in *Tethya lyncurium* [118]. Similarly, the buds of *T. sychellensis* contain a dense collagen matrix [119]. Buds consist of cellular masses that sprout out from the surface of adults and are able to develop into new functional individuals [119].

Recently, special attention has been focused on fibrillar collagens in the mesohyl of *C. reniformis*. This species is the only sponge which has been experimentally proven to contain a dynamic collagenous mesohyl capable of stiffening upon being manipulated [120]. It was shown that the different physiological states recorded in laboratory experiments are expressions of the mechanical adaptability of the collagenous mesohyl of *C. reniformis*, and suggest that stiffness variability in this sponge is under cellular control [121].

## 5. *Chondrosia* Collagens

Collagens from the demosponge *Chondrosia reniformis* (Nardo 1847) have received attention from researchers since 1970 [113,117,118,119] due to their diversity (type IV collagen, fibrillar and nonfibrillar collagens) [120] and interesting structural [121], physicochemical [122], and ecophysiological properties [123,124,125,126,127]. For example, slices of fibrillar collagen incubated with collagenase are not modified even after 48 h of incubation, and do not show any changes in the aspect, consistency, or fine structure of the fibrils. No kind of enzymatic damage was observed by electron microscope on the isolated collagen fibrils after collagenase treatment.

**Figure 7:**
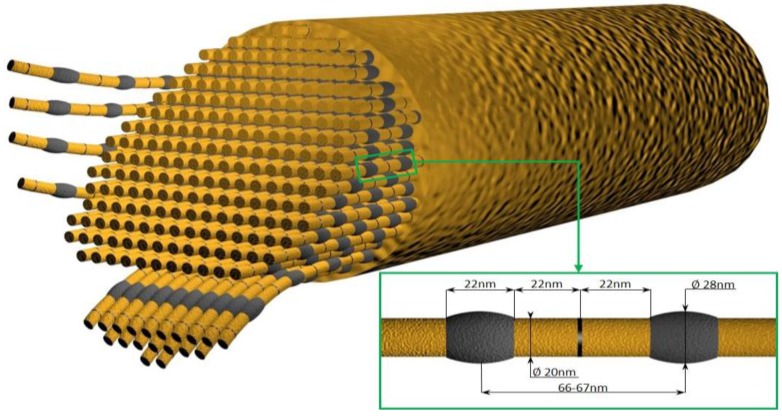
Schematic diagram of *C. reniformis* collagen fiber with numerous nanofibrils with characteristic nanotopography. Along the fibril, one characteristically thick segment (protrusion) about 28 nm in diameter is followed by two equal thinner and closer conjoined segments (interband) about 20 nm in diameter. The average distance between the protrusions is about 67–69 nm. The distance between two consecutive peaks of the interband regions or between a protrusion and an adjacent interband region is about 21–23 nm. The average step height between the protrusions and the interband regions was calculated to be about 4 nm (see for review [121]).

The mechanical properties of this collagen have been partially described by Garrone et al. [117]. The cortex of *Chondrosia* sponges is less resistant than calf skin, but has mechanical properties of the same order as those of bovine nasal cartilage (Young’s modulus 150–250 kg/cm^2^ and 100–250 kg/cm^2^ respectively). Probably due to special mechanical features, the body of *Chondrosia* can slowly become flat and slide to avoid compression or stretch itself into a slender thread under continuous stress. Such *creeping* behavior of a fibrous and living material provides a remarkable example for the study of mechanical stresses as morphogenetic factors [117]. Although the nanomorphology of *C. reniformis* collagen fibrils has now been well investigated (Figure 7) [121], there is still a lack of knowledge about the relationship between the ultrastructural features of this collagen and its mechanical and physicochemical properties.

The well-known biocompatibility of *C. reniformis* fibrillar collagen has stimulated many studies on its possible applications in cosmetics and pharmacology (see for review [31]), including in transdermal drug delivery [128].

The production and selection of a triple transformed *Pichia pastoris* yeast strain expressing a stable P4H tetramer derived from *C. reniformis* sponge and a hydroxylated nonfibrillar procollagen polypeptide from the same organism have recently been reported by Giovine et al. [118]. The obtained recombinant sponge P4H has the ability to hydroxylate its natural substrate in both X and Y positions in the Xaa-Yaa-Gly collagenous triplets. It is suggested that the *Pichia* system could be used for the large-scale production of hydroxylated sponge- or marine-derived collagen polypeptides, which have high pharmacological potential [118].

The possibility of the application of *Chondrosia* fibrillar collagen as an organic template for in vitro silicification has been confirmed in several studies [121,129,130]. There are no doubts that the mechanical properties of biomimetically-inspired hybrid composites can be significantly improved with the presence of this special collagen.

## 6. Glass Sponge Collagen

Collagen is known as a universal template in biomineralization, including both calcification and silicification. It is proposed that this biopolymer functions as a fundamental template in biomineralization, inasmuch as it is very ancient from an evolutionary point of view and is common to many species and biological systems with a global distribution [131]. The identification of diverse collagens in demosponges as described above suggests that they may also be found within skeletal structures in the sister group, the glass sponges. Hexactinellida Schmidt (Porifera), with more than 700 species, consists exclusively of marine glass sponges. These are psychrophilic organisms which can produce huge biosilica-based skeletons and anchoring spicules at temperatures between −2 °C and 4 °C [132].

**Figure 8:**
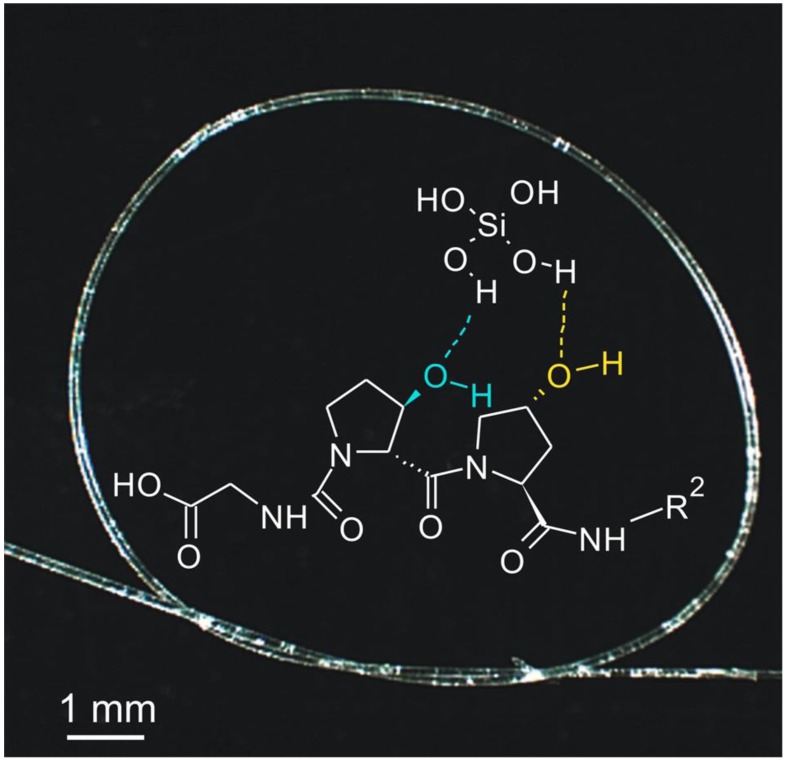
Photograph demonstrating the unique flexibility of the *H. sieboldi* anchoring spicule, and schematic view of the role of special hydroxylated collagen in silica condensation in this natural basilica structure (for details see [3]).

**Figure 9:**
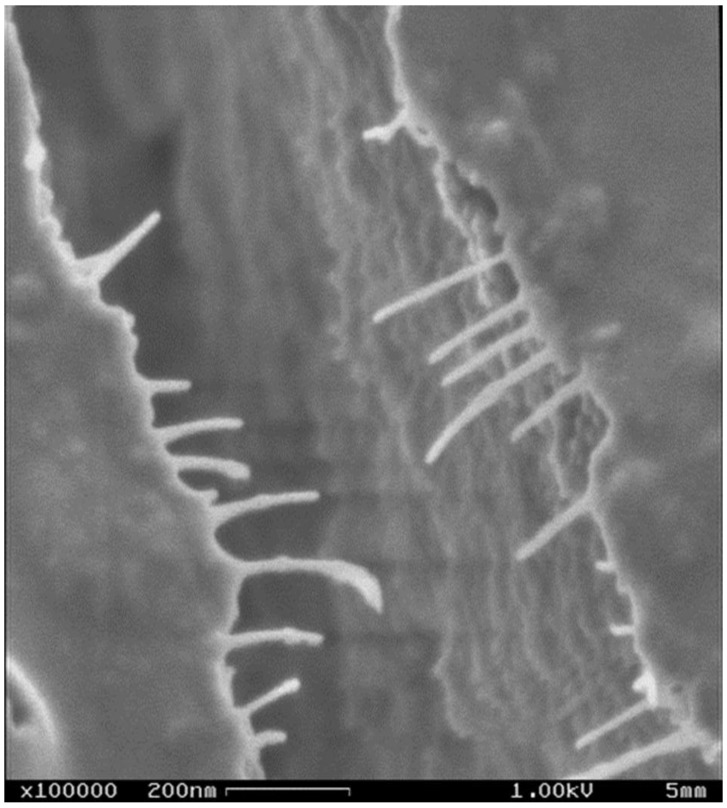
SEM image of the nanofibrillar collagenous layer on the surface of an *H. sieboldi* glass sponge anchoring spicule.

The challenging task of isolating and identifying collagen in the skeletal structures of diverse glass sponges was completed successfully only in 2010 [3], following numerous attempts at gentle demineralization [133,134]. Studies in this area have been motivated by the great flexibility of the glassy spicules, which allows researchers to tie a spicule into a bundle (Figure 8). It has been suggested that this peculiar feature of spicules in the hexactinellids must be due to the presence of a structural carcass of organic nature both on the surface (Figure 9) and within the spicules [133].

The organic phase has been identified as a highly hydroxylated fibrillar collagen which contains an unusual [Gly–3Hyp–4Hyp] motif predisposed for silica precipitation, and provides a novel template for biosilicification in nature [3]. This collagen presents a layer of hydroxyl groups that can undergo condensation reactions with silicic acid molecules with consequent loss of water. As a result, the initial layer of condensed silicic acid will be held fixed to the collagenous template in a geometric arrangement that will favor further polymerization of silicic acid. It therefore appears that collagen was a novel template for biosilicification that emerged at an early stage during metazoan evolution, and that the occurrence of additional trans-3-Hyp plays a key role in stabilizing silicic acid molecules and initiating the precipitation of silica.

**Figure 10:**
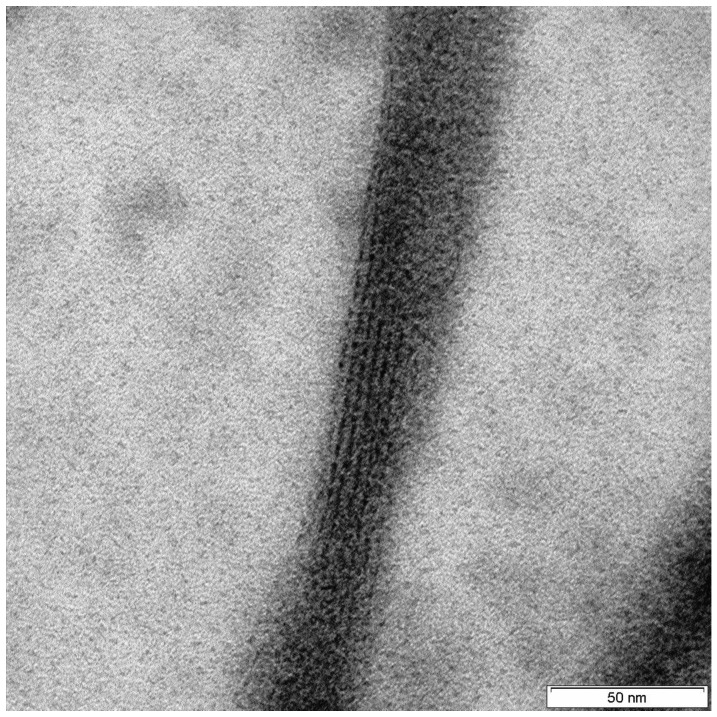
High-resolution transmission electron microscope image of a fragment of *M. chuni* collagen nanofibril isolated from the glassy spicule (for details see [135]). The nanomorphology of such fibrils is similar to that from *H. sieboldi* glass sponge collagen [3], but different from the striated collagen fibrils from the demosponge *C. reniformis* [121].

Collagen has also been reported as the main organic component of the spicules of the glass sponge *Monorhaphis* sp. [135] (Figure 10). Results of the amino acid analysis of protein extracts isolated from demineralized spicules of this sponge showed an amino acid content typical for collagens of the same origin. Comparison with the Microsatellite Database (MSDB) protein database led to the identification of alpha 1 collagen in two high-MW bands. In contrast to its analog in *H. sieboldi*, collagen isolated and identified from *Monorhaphis* sp. was matched only to the type I collagen pre-pro-alpha (I) chain (COL1A1) from dog (AAD34619) (MW 139,74) [135].

The existence of naturally occurring collagen–silica-based composites in the form of spicules of glass sponges stimulated material scientists to develop analogous hybrid materials. Due to the limited available amounts of glass sponge collagen for the development of silica-based composite materials, fibrillar collagen from the demosponge *C. reniformis* has been successfully used as an alternative by the Ehrlich research group [121,129]. More recently, a new concept in biosilica material synthesis which does not require phosphate supplements and is based on the fusion of stabilized polysilicic acid into a fluidic precursor phase upon infiltration into polyamine-enriched collagen has been proposed by the Tay research group [136,137,138]. It has recently been shown that silicified collagen scaffolds produced by infiltrating collagen matrices with intrafibrillar amorphous silica exhibit angiogenic and osteogenic potential and can be used in tissue engineering [139]. In work by Aime et al. [140], collagen triple helices have been confined on the surface of sulfonate-modified silica particles in a controlled manner. This gives rise to hybrid building blocks with well-defined surface potentials and dimensions. Additionally, oligomeric collagen-fibril matrices with tunable microstructural properties have been used to template and direct the formation of biocompatible mesoporous sol–gel silica to develop nanostructured hybrid organic–inorganic composites [141]. It was experimentally confirmed that silica mineralization kinetics are critical for the precision-tuning of properties of the hybrid materials, including porous microstructure, mechanical strength, depth of silica penetration, and mass transport properties. It has also been shown that microstructural properties of the collagen-fibril template are preserved in the silica surface of hybrid materials [142]. Such novel silica-collagen hybrid materials may be useful, for example, in the regeneration of bone tissue or in cellular microencapsulation [141].

## 7. Conclusions

We have presented here only a brief discussion of selected examples, which nonetheless provide novel data concerning poriferan collagens. In spite of the progress made in this research field, numerous open questions remain. For example, additional investigations are necessary to obtain understanding of the nature and origin of halogenated spongins. It is still not clear how many collagen and/or keratin domains they contain. Additionally, the unique resistance of these biopolymers against diverse chemicals and enzymes remains poorly investigated. The possible role of collagens in the spiculogenesis of demosponges and formation of axial filaments must also be researched. The discovery of crystalline proteins within amorphous biosilica-based structures in sponges is ground-breaking in the understanding of biomineralization. What can be discovered about the crystallinity of collagen within biosilica-related structures in sponges? The existence of collagen-based crystals within siliceous biominerals could revolutionize our understanding of the origin and evolution of collagens, from the point of view of biomineralization in sponges as the first multicellular organisms on Earth. Further, the relationship of collagen and chitin in the skeletal structures of diverse sponge classes and orders is entirely unknown. Consequently, we believe that the use of modern X-ray imaging techniques based on the “diffraction before destruction” principle is the best way forward to gain understanding of the principles of the unique organization of collagen within both fossil and recent collagen-based biomineralized constructs.

Novel approaches must be proposed which will bring together modern bioanalytical and molecular biology methods for better understanding of the fundamental principles of collagen fibrillogenesis and the mechanisms of its cross-linking in sponges, as well as details of the structural organization of poriferan collagens at the molecular and atomic levels. The best way to address this challenging task on these levels is by coherent synergetic collaboration using explicit reasoning and well-tested explanatory principles of multidisciplinary knowledge, experience, and new technologies. Finally, we suggest that studying the processes of marine farming of the collagen-producing demosponges has implications for a variety of practical large-scale applications, ranging from the design of highly effective extraction techniques to the development of novel collagen-containing composites for biomedicine and technology.

## Figures and Tables

**Table 1:** Amino acid composition of spongin.

Constituent	Content (%)
Nitrogen	13.0–14.8
Sulfur	0.7
Iodine	0.84–1.46
Histidine	0–0.2
Lysine	3–3.6
Arginine	4.3–5.9
Cystine	2.8
Tyrosine	0–0.8
Tryptophan	0
Phenylalanine	3.3
Glycine	13.9–14.4
Diiodotyrosine	4.7
Molecular ratio of lysine to arginine	4:6

**Table 2:** The chemistry of spongin according to Ackerman and Burkhard [69].

C	H	N	I	Br	S	Cl	Ashes
47.00	6.28	16.06	1.41	2.93	0.87	0	1.16

## References

[1] Luo Y., Sinkeviciute D., He Y., Karsdal M., Henrotin Y., Mobasheri A., Önnerfjord P., Bay-Jensen A. (2017). The minor collagens in articular cartilage. Protein Cell.

[2] Tzaphlidou M., Berillis P. (2002). Structural alterations caused by lithium in skin and liver collagen using an image processing method. J. Trace Microprobe Tech..

[3] Ehrlich H., Deutzmann R., Brunner E., Cappellini E., Koon H., Solazzo C., Yang Y., Ashford D., Thomas-Oates J., Lubeck M. (2010). Mineralization of the metre-long biosilica structures of glass sponges is templated on hydroxylated collagen. Nat. Chem..

[4] Shahar R., Weiner S. (2018). Open questions on the 3D structures of collagen containing vertebrate mineralized tissues: A perspective. J. Struct. Biol..

[5] Cai J., Palamara J.E.A., Burrow M.F. (2018). Effects of collagen crosslinkers on dentine: A literature review. Calcif. Tissue Int..

[6] Ramachandran G.N., Kartha G. (1955). Structure of collagen. Nature.

[7] Rich A., Crick F.H.C. (1955). The structure of collagen. Nature.

[8] Shoulders M.D., Raines R.T. (2009). Collagen structure and stability. Annu. Rev. Biochem..

[9] Squire J.M., Parry D.A. (2017). Fibrous protein structures: Hierarchy, history and heroes. Subcell. Biochem..

[10] Bella J., Hulmes D.J. (2017). Fibrillar collagens. Subcell. Biochem..

[11] Ricard-Blum S. (2011). The collagen family. Cold Spring Harb. Perspect. Biol..

[12] Porfírio E., Fanaro G.B. (2016). Collagen supplementation as a complementary therapy for the prevention and treatment of osteoporosis and osteoarthritis: A systematic review. Rev. Bras. Geriatr. Gerontol..

[13] Pawelec K.M., Best S.M., Cameron R.E. (2016). Collagen: A network for regenerative medicine. J. Mater. Chem. B.

[14] Conway J.R.W., Vennin C., Cazet A.S., Herrmann D., Murphy K.J., Warren S.C., Wullkopf L., Boulghourjian A., Zaratzian A., Da Silva A.M. (2017). Three-dimensional organotypic matrices from alternative collagen sources as pre-clinical models for cell biology. Sci. Rep..

[15] Avila Rodríguez M.I., Rodríguez Barroso L.G., Sánchez M.L. (2018). Collagen: A review on its sources and potential cosmetic applications. J. Cosmet. Dermatol..

[16] Hashim P., Mohd Ridzwan M.S., Bakar J., Hashim M. (2015). Collagen in food and beverage industries. Int. Food Res. J..

[17] Raspanti M., Requzzoni M., Protasoni M., Basso P. (2018). Not only tendons: The other architecture of collagen fibrils. Int. J. Biol. Macromol..

[18] Uzel S.G., Buehler M.J. (2009). Nanomechanical sequencing of collagen: Tropocollagen features heterogeneous elastic properties at the nanoscale. Integr. Biol..

[19] Ehrlich H. (2015). Biological Materials of Marine Origin. Vertebrates.

[20] Alves A.L.P., Marques A.L., Martins E., Silva T.H., Reis R.L. (2017). Cosmetic potential of marine fish skin collagen. Cosmetics.

[21] Venkatesan J., Anil S., Kim S.K., Shim M.S. (2017). Marine fish proteins and peptides for cosmeceuticals: A review. Mar. Drugs.

[22] Berillis P., Saxena M. (2015). Marine Collagen: Extraction and applications. Research Trends in Biochemistry, Molecular Biology and Microbiology.

[23] Zhang J., Sun Y., Zhao Y., Wei B., Xu C., He L., Oliveira C.L.P., Wang H. (2017). Centrifugation-induced fibrous orientation in fish-sourced collagen matrices. Soft Matter.

[24] Adams E. (1978). Invertebrate collagens. Science.

[25] Bailey A.F., Florkin M., Stotz E.H. (1968). The nature of collagen. Comprehensive Biochemistry, Extracellular and Supporting Structures.

[26] Engel J. (1997). Versatile collagens in invertebrates. Science.

[27] Exposito J.Y., Cluzel C., Garrone R., Lethias C. (2002). Evolution of collagens. Anat. Rec..

[28] Garrone R. (1999). Evolution of metazoan collagens. Prog. Mol. Subcell. Biol..

[29] Gross J., Sokal Z., Rougvie M. (1956). Structural and chemical studies on the connective tissue of marine sponges. J. Histochem. Cytochem..

[30] Tanzer M.L. (1978). The biological diversity of collagenous proteins. Trends Biochem. Sci..

[31] Silva T.H., Moreira-Silva J., Marques A.L.P., Domingues A., Bayon Y., Reis R.L. (2014). Marine origin collagens and its potential applications. Mar. Drugs.

[32] Goh K.L., Holmes D.F. (2017). Collagenous extracellular matrix biomaterials for tissue engineering: Lessons from the common sea urchin tissue. Int. J. Mol. Sci..

[33] Betancourt-lozano M., Gonzalez-farias F., Garcıa-gasca A. (1998). Variation of antimicrobial activity of the sponge *Aplysina fistularis* (Pallas, 1766) and its relation to associated fauna. J. Exp. Mar. Biol. Ecol..

[34] Pallela R., Ehrlich H. (2016). Marine Sponges: Chemicobiological and Biomedical Applications.

[35] Reitner J., Mehl D. (1996). Monophyly of the Porifera. Verh. Naturwiss. Ver. Hambg..

[36] Nichols S., Wörheide G. (2005). Sponges: New views of old animals. Integr. Comp. Biol..

[37] Moldowan J.M. (1984). C_30_ steranes, novel markers for marine petroleums and sedimentary rocks. Geochim. Cosmochim. Acta.

[38] Moldowan J.M., Seifert W.K., Gallegos E.J. (1985). Relationship between petroleum composition and depositional environment of petroleum source rocks. Am. Assoc. Pet. Geol. Bull..

[39] Moldowan J.M., Fago F.J., Lee C.Y., Jacobson S.R., Watt D.S., Slougui N.-E., Jeganathan A., Young D.C. (1990). Sedimentary 24+propylcholestanes, molecular fossils diagnostic of marine algae. Science.

[40] Wainright P.O., Hinkle G., Sogin M.L., Stickel S.K. (1993). Monophyletic origins of the Metazoa: An evolutionary link with fungi. Science.

[41] Wood R. (1990). Reef-building sponges. Am. Sci..

[42] Van Soest R.W.M., Boury-Esnault N., Vacelet J., Dohrmann M., Erpenbeck D., de Voogd N.J., Santodomingo N., Vanhoorne B., Kelly M., Hooper J.N.A. (2012). Global diversity of sponges (Porifera). PLoS ONE.

[43] Garrone R. (1978). Phylogenesis of Connective Tissue: Morphological Aspects and Biosynthesis of Sponge Intercellular Matrix.

[44] Junqua S., Robert L., Garrone R. (1974). Biochemical and morphological studies on the collagens of horny sponges. *Ircinia* filaments compared to spongines. Connect. Tissue Res..

[45] Städeler G. (1859). Untersuchungen über das Fibroin, Spongin und Chitin, nebst Bemerkungen über den tierischen Schleim. Eur. J. Organ. Chem..

[46] Green D., Howard D., Yang X., Kelly M., Oreffo R.O.C. (2003). Natural marine sponge fiber skeleton: A biomimetic scaffold for human osteoprogenitor cell attachment, growth, and differentiation. Tissue Eng..

[47] Green D.W., Lee J.-M., Jung H.-S. (2015). Marine structural biomaterials in medical biomimicry. Tissue Eng. Part B Rev..

[48] Green D.W., Padula M.P., Santos J., Chou J., Milthorpe B., Ben-Nissan B. (2013). A therapeutic potential for marine skeletal proteins in bone regeneration. Mar. Drugs.

[49] Lin Z., Solomon K.L., Zhang X., Pavlos N.J., Abel T., Willers C. (2011). In vitro evaluation of natural marine sponge collagen as a scaffold for bone tissue engineering. Int. J. Biol. Sci..

[50] Pallela R., Venkatesan J., Bhatnagar I., Shim Y., Kim S., Kim S.-K. (2011). Application of marine collagen–based scaffolds in bone tissue engineering. Marine Biomaterials: Isolation, Characterization and Applications.

[51] Nandi S.K., Kundu B., Mahato A., Thakur N.L., Joardar S.N., Mandal B.B. (2015). In vitro and in vivo evaluation of the marine sponge skeleton as a bone mimicking biomaterial. Integr. Biol..

[52] Langasco R., Cadeddu B., Formato M., Lepedda A.J., Cossu M., Giunchedi P., Pronzato R., Rassu G., Manconi R., Gavini R. (2017). Natural collagenic skeleton of marine sponges in pharmaceutics: Innovative biomaterial for topical drug delivery. Mater. Sci. Eng. C.

[53] Tziveleka L.A., Ioannou E., Tsiourvas D., Berillis P., Foufa E., Roussis V. (2017). Collagen from the marine sponges *Axinella cannabina* and *Suberites carnosus*: Isolation and morphological, biochemical, and biophysical characterization. Mar. Drugs.

[54] De Laubenfels M., Storr J. (1958). The taxonomy of American commercial sponges. Bull. Mar. Sci..

[55] Schulze F.E. (1877). Untersuchungen über den Bau und die Entwicklung der Spongien. Z. wiss. Zool..

[56] Cresswell E. (1922). Sponges: Their Nature, History, Modes of Fishing, Varieties, Cultivation, etc..

[57] Geoffroy C.J. (1705). Analyse chimique de l’eponge de la moyenne espece. Hist. Acad. R. Sci..

[58] Fyfe A. (1819). Account of some experiments, made with the view of ascertaining the different substances from which iodine can be produced. Edinb. Philos..

[59] Croockewit J.H. (1843). Ueber die Zusammensetzung des Badeschwammes. Eur. J. Organ. Chem..

[60] Schlossberger J. (1859). Ueber Fibroin und die Substanz des Badeschwamms. Arch. Pharm..

[61] Von Kölliker A. (1864). Der feinere Bau der Protozoen.

[62] Hundeshagen F. (1895). Über jodhaltige Spongien and Jodospongin. Angew. Chem. Int. Ed..

[63] Harnack E. (1898). Ueber das iodospongin, die jodhaltige eiweissartige Subsanz aus dem Badeschwamm. Z. Physiol. Chem..

[64] Clancey V.J. (1926). The constitution of sponges. The common bath sponge, *Hippospongia equina*. Biochem. J..

[65] Abderhalden E., Strauss E. (1906). Die Spaltprodukte des Spongins mit Sauren. Hoppe-Seyler’s Z. Physiol. Chem..

[66] Strauss E. (1904). Studien ueber die Albuminoide mit Besonderer Berucksichtigung des Spongins und der Keratine.

[67] Jenkins C.L. (2002). Insights on the conformational stability of collagen. Nat. Prod. Rep..

[68] Block R.J., Bolling D. (1939). The amino acid composition of keratins. J. Biol. Chem..

[69] Ackermann D., Burchard C. (1941). Zur Kenntnis der Spongine. Hoppe-Seyler’s Z. Physiol. Chem..

[70] Ackermann D., Müller I. (1941). Über das Vorkommen von Dibromotyrosin neben Dijodtyrosin im Spongin. Hoppe-Seyler’s Z. Physiol. Chem..

[71] Low E.M. (1951). Halogenated amino acids of the bath sponge. J. Mar. Res..

[72] Katzman R.L., Halford M.H., Reinhold V.N., Jeanloz R.W. (1972). Invertebrate connective tissue. IX. Isolation and structure determination of glucosylgalactosylhydroxylysine from sponge and sea anemone collagen. Biochemistry.

[73] Gaino E., Pronzato R. (1989). Ultrastructural evidence of bacterial damage to *Spongia officinalis* fibres (Porifera, Demospongiae). Dis. Aquat. Organ..

[74] Ehrlich H., Maldonado M., Spindler K., Eckert C., Hanke T., Born R., Simon P., Heinemann S., Worch H. (2007). First evidence of chitin as a component of the skeletal fibers of marine sponges. Part I. Verongida (Demospongia: Porifera). J. Exp. Zool. Part B.

[75] Ehrlich H., Ilan M., Maldonado M., Muricy G., Bavestrello G., Kljajic Z., Carballo J.L., Schiaparelli S., Ereskovsky A., Schupp P. (2010). Three-dimensional chitin-based scaffolds from Verongida sponges (Demospongiae: Porifera). Part I. Isolation and identification of chitin. Int. J. Bol. Macromol..

[76] Wysokowski M., Bazhenov V.V., Tsurkan M.V., Galli R., Stelling A.L., Stöcker H., Kaiser S., Niederschlag E., Gärtner G., Behm T. (2013). Isolation and identification of chitin in three-dimensional skeleton of *Aplysina fistularis* marine sponge. Int. J. Biol. Macromol..

[77] Ehrlich H., Kaluzhnaya O.V., Tsurkan M.V., Ereskovsky A., Tabachnick K.R., Ilan M., Stelling A., Galli R., Petrova O.V., Nekipelov S.V. (2013). First report on chitinous holdfast in sponges (Porifera). Proc. R. Soc. Lond. B.

[78] Ehrlich H., Kaluzhnaya O.V., Brunner E., Tsurkan M.V., Ereskovsky A., Ilan M., Tabachnick K.R., Bazhenov V.V., Paasch S., Kammer M. (2013). Identification and first insights into the structure and biosynthesis of chitin from the freshwater sponge *Spongilla lacustris*. J. Struct. Biol..

[79] Wysokowski M., Petrenko I., Stelling A., Stawski D., Jesionowski T., Ehrlich H. (2015). Poriferan chitin as a versatile template for extreme biomimetics. Polymers.

[80] Shavandi A., Silva T.H., Bekhit A.A., Bekhit A.E. (2017). Keratin: Dissolution, extraction and biomedical application. Biomater. Sci..

[81] Cerrano C., Calcinai B., Gioia C., Camillo D., Valisano L., Bavestrello G., Custódio M.R., Lôbo-Hajdu G., Hajdu E., Muricy G. (2007). How and why do sponges incorporate foreign material? Strategies in porifera. Porifera Research: Biodiversity, Innovation and Sustainability.

[82] Castritsi-Catharios J., Zaoutsos S.P., Berillis P., Zouganelis G.D., Ekonomou G., Kefalas E., Pantelis J. (2017). Kalymnos, the island which made history in sponge fishery. Data on physical parameters, elemental composition and DNA barcode preliminary results of the most common bath sponge species in Aegean Sea. Reg. Stud. Mar. Sci..

[83] Castritsi-Catharios J., Zaoutsos S.P., Ekonomou G., Berillis P. Physical parameters and chemical composition of four sponge species. Preliminary results. Proceedings of the HydroMedit 2016.

[84] Castritsi-Catharios J., Magli M., Vacelet J. (2007). Evaluation of the quality of two commercial sponges by tensile strength measurement. J. Mar. Biol. Assoc. UK.

[85] Dandy A. (1916). On the occurrence of gelatinous spicules, and their mode of origin, in a new genus of siliceous sponges. Proc. R. Soc. Lond. B.

[86] Szatkowski T., Jesionowski T., Ehrlich H. (2017). Hydrothermal synthesis of spongin-based materials. Extreme Biomimetics.

[87] Von Raimann E. (1939). Über den Nutzen des Bade- oder Waschschwammes bey heftigen Blutun-gen. Med. Jahrbücher.

[88] White C. (1770). An account of the successful use of the sponge, in the stoppage of an Haemorrhage, occasioned by amputation below the knee; and of the remarkable effects of that application in preventing the absorption of matter. Cases in Surgery with Remarks: Part 1.

[89] Zschiesche P. (1873). Die Anwendung des Pressschwammes in der Gynaekologie und Seine Gefahren.

[90] Haussmann D. (1878). Kann die Erweiterung des Verengten Muttermundes durch Pressschwamm die Empfangniss erleichtern?.

[91] Petrus C. (1771). Naauwkeurige Afbelding en Beschryving van eene Geheel en al Verloorene, Maar door Kunst Herstelde Neus en Verhemelte.

[92] Hamilton D.J. (1881). On sponge-grafting. Edinb. Med. J..

[93] Kim M.M., Mendis E., Rajapakse N., Lee S.H., Kim S.K. (2009). Effect of spongin derived from *Hymeniacidon sinapium* on bone mineralization. J. Biomed. Mater. Res. B.

[94] Norman M., Bartczak P., Zdarta J., Ehrlich H. (2016). Anthocyanin dye conjugated with *Hippospongia communis* marine demosponge skeleton and its antiradical activity. Dyes Pigments.

[95] Norman M., Bartczak P., Zdarta J., Tomala W., Żurańska B., Dobrowolska A., Piasecki A., Czaczyk K., Ehrlich H., Jesionowski T. (2016). Sodium copper chlorophyllin immobilization onto *Hippospongia communis* marine demosponge skeleton and its antibacterial activity. Int. J. Mol. Sci..

[96] Zdarta J., Norman M., Smułek W., Moszyński D., Kaczorek E., Stelling A.L., Ehrlich H., Jesionowski T. (2017). Spongin-based scaffolds from *Hippospongia communis* demosponge as an effective support for lipase immobilization. Catalysts.

[97] Szatkowski T., Siwińska-Stefańska K., Wysokowski M., Stelling A., Joseph Y., Ehrlich H., Jesionowski T. (2017). Immobilization of titanium(IV) oxide onto 3D spongin scaffolds of marine sponge origin according to extreme biomimetics principles for removal of C.I. Basic Blue 9. Biomimetics.

[98] Szatkowski T., Wysokowski M., Lota G., Pęziak D., Bazhenov V.V., Nowaczyk G., Walter J., Molodtsov S.L., Stöcker H., Himcinschi C. (2015). Novel nanostructured hematite–spongin composite developed using an extreme biomimetic approach. RSC Adv..

[99] Ehrlich H. (2017). Extreme Biomimetics.

[100] Chioran A., Duncan S., Catalano A., Brown T.J., Ringuette M.J. (2017). Collagen IV trafficking: The inside-out and beyond story. Dev. Biol..

[101] Fidler A.L., Darris C.E., Chetyrkin S.V., Pedchenko V.K., Boudko S.P., Brown K.L., Gray Jerome W., Hudson J.K., Rokas A., Hudson B.G. (2017). Collagen IV and basement membrane at the evolutionary dawn of metazoan tissues. eLife.

[102] Rodriguez-Pascual F., Slatter D.A. (2016). Collagen cross-linking: Insights on the evolution of metazoan extracellular matrix. Sci. Rep..

[103] Boute N., Exposito J.Y., Boury-Esnault N., Vacelet J., Nor N., Miyazaki K., Yoshizato K., Garrone R. (1996). Type IV collagen in sponges, the missing link in basement membrane ubiquity. Biol. Cell.

[104] Leys S.P., Riesgo A. (2011). Epithelia, an evolutionary novelty of metazoans. J. Exp. Zool. Part B.

[105] Riesgo A., Taboada S., Sánchez-Vila L., Solà J., Bertran A., Avila C. (2015). Some like it fat: Comparative ultrastructure of the embryo in two demosponges of the genus Mycale (order Poecilosclerida) from Antarctica and the Caribbean. PLoS ONE.

[106] Exposito J.Y., Le Guellec D., Lu Q., Garrone R. (1991). Short chain collagens in sponges are encoded by a family of closely related genes. J. Biol. Chem..

[107] Aouacheria A., Geourjon C., Aghajari N., Navratil V., Deléage G., Lethias C., Exposito J.Y. (2006). Insights into early extracellular matrix evolution: Spongin short chain collagen-related proteins are homologous to basement membrane type IV collagens and form a novel family widely distributed in invertebrates. Mol. Biol. Evol..

[108] Rocha Moreira Da Silva J.C., Soares Diogo Carlos G., De Sousa E Silva Barros Prata M., Quinteiros Lopes Henriques Da Silva T.J., Pinto Marques A.M., Gonçalves Dos Reis R.L., Teixeira Cerqueira M., Vieira Pereira Ferreira M.S. (2015). Marine-Sponge Type IV Collagen Membranes Its Production and Biomedical Applications Thereof. Patent.

[109] Cavalier-Smith T. (2017). Origin of animal multicellularity: Precursors, causes, consequences—The choanoflagellate/sponge transition, neurogenesis and the Cambrian explosion. Philos. Trans. R. Soc. Lond. B.

[110] Simpson T.L. (1984). Collagen Fibrils, Spongin, Matrix Substances. The Cell Biology of Sponges.

[111] Garrone R., Pottu J. (1973). Collagen biosynthesis in sponges—Elaboration of spongin by spongocytes. J. Submicrosc. Cytol..

[112] Bairati A., Garrone R. (1985). Biology of Invertebrate and Lower Vertebrate Collagens.

[113] Imhoff J.M., Garrone R. (1983). Solubilization and characterization of *Chondrosia reniformis* sponge collagen. Connect. Tissue Res..

[114] Eckert C., Schröder H.C., Brandt D., Perovic-Ottstadt S., Muller W.E.G. (2006). Histochemical and electron microscopic analysis of spiculogenesis in the demosponge *Suberites domuncula*. Zootaxa.

[115] Ehrlich H. (2010). Biological Materials of Marine Origin. Invertebrates.

[116] Krasko A., Lorenz B., Batel R., Schröder H.C., Müller I.M., Müller W.E.G. (2000). Expression of silicatein and collagen genes in the marine sponge *Suberites domuncula* is controlled by silicate and myotrophin. Eur. J. Biochem..

[117] Garrone R., Huc A., Junqua S. (1975). Fine structure and physicochemical studies on the collagen of the marine sponge *Chondrosia reniformis* Nardo. J. Ultrastruct. Res..

[118] Pozzolini M., Scarfi S., Mussino F., Ferrando S., Gallus L., Giovine M. (2015). Molecular cloning, characterization, and expression analysis of a prolyl 4-hydroxylase from the marine sponge *Chondrosia reniformis*. Mar. Biotechnol..

[119] Ledger P.W. (1974). Types of collagen fibres in the calcareous sponges *Sycon* and *Leucandra*. Tissue Cell.

[120] Pozzolini M., Bruzzone F., Berilli V., Mussino F., Cerrano C., Benatti U., Giovine M. (2012). Molecular characterization of a nonfibrillar collagen from the marine sponge *Chondrosia reniformis* Nardo 1847 and positive effects of soluble silicates on its expression. Mar. Biotechnol..

[121] Heinemann S., Ehrlich H., Douglas T., Heinemann C., Worch H., Schatton W., Hanke T. (2007). Ultrastructural studies on the collagen of the marine sponge *Chondrosia reniformis* Nardo. Biomacromolecules.

[122] Palmer I., Clarke S.A., Nelson J., Schatton W., Dunne N.J., Buchanan F. (2012). Identification of a suitable sterilisation method for collagen derived from a marine Demosponge. Int. J. Nano Biomater..

[123] Bavestrello G., Cerrano C., Cattaneo-Vietti R., Sara M., Calabria F., Cortesogno L. (1996). Selective incorporation of foreign material in *Chondrosia reniformis* (Porifera, Demospongiae). Ital. J. Zool..

[124] Bavestrello G., Benatti U., Calcinai B., Cattaneo-Vietti R., Cerrano C., Favre A., Giovine M., Lanza S., Pronzato R., Sara M. (1998). Body polarity and mineral selectivity in the demosponge *Chondrosia reniformis*. Biol. Bull..

[125] Fassini D. (2013). Coordination Phenomena in the Marine Demosponge Chondrosia reniformis.

[126] Fassini D., Parma L., Lembo F., Candia Carnevali M., Wilkie I., Bonasoro F. (2014). The reaction of the sponge *Chondrosia reniformis* to mechanical stimulation is mediated by the outer epithelium and the release of stiffening factor(s). Zoology.

[127] Pozzolini M., Ferrando S., Gallus L., Gambardella C., Ghignone S., Giovine M. (2016). Aquaporin in *Chondrosia reniformis* Nardo, 1847 and its possible role in the interaction between cells and engulfed siliceous particles. Biol. Bull..

[128] Nicklas M., Schatton W., Heinemann S., Hanke T., Kreuter J. (2009). Preparation and characterization of marine sponge collagen nanoparticles and employment for the transdermal delivery of 17β-estradiol-hemihydrate. Drug Dev. Ind. Pharm..

[129] Heinemann S., Heinemann C., Ehrlich H., Meyer M., Baltzer H., Worch H., Hanke T. (2007). A novel biomimetic hybrid material made of silicified collagen: Perspectives for bone replacement. Adv. Eng. Mater..

[130] Heinemann S., Ehrlich H., Knieb C., Hanke T. (2007). Biomimetically inspired hybrid materials based on silicified collagen. Int. J. Mater. Res..

[131] Ehrlich H. (2010). Chitin and collagen as universal and alternative templates in biomineralization. Int. Geol. Rev..

[132] Tabachnick K., Janussen D., Menschenina L., Ehrlich H. (2017). Cold biosilicification in Metazoan: Psychrophilic glass sponges. Extreme Biomimetics.

[133] Ehrlich H., Ereskovskii V., Drozdov L., Krylova D.D., Hanke T., Meissner H., Heinemann S., Worch H. (2006). A modern approach to demineralization of spicules in glass sponges (Porifera: Hexactinellida) for the purpose of extraction and examination of the protein matrix. Russ. J. Mar. Biol..

[134] Ehrlich H., Worch H., Custodio M.R., Lobo-Hajdu G., Hajdu E., Muricy G. (2007). Sponges as natural composites. Porifera Research Biodiversity, Innovation and Sustainability.

[135] Ehrlich H., Heinemann S., Heinemann C., Simon P., Bazhenov V.V., Shapkin N.P., Born R., Tabachnick K.R., Hanke T., Worch H. (2008). Nanostructural organization of naturally occurring composites—Part I: Silica-collagen-based biocomposites. J. Nanomater..

[136] Niu L., Jiao K., Qi Y., Yiu C.K.Y., Ryou H., Arola D.D., Chen J., Breschi L., Pashley D.H., Tay F.R. (2011). Infiltration of silica inside fibrillar collagen. Angew. Chem. Int. Ed..

[137] Luo X.J., Yang H.Y., Niu L.N., Mao J., Huang C., Pashley D.H., Tay F.R. (2016). Translation of a solution-based biomineralization concept into a carrier-based delivery system via the use of expanded-pore mesoporous silica. Acta Biomater..

[138] Zhang W., Luo X., Niu L., Yang H., Yiu C.K., Wang T., Zhou L., Mao J., Huang C., Pashley D.H., Tay F.R. (2015). Biomimetic intrafibrillar mineralization of type I collagen with intermediate precursors-loaded mesoporous carriers. Sci. Rep..

[139] Sun J.-L., Jiao K., Niu L.N., Jiao Y., Song Q., Shen L.J., Tay F.R., Chen J.H. (2017). Intrafibrillar silicified collagen scaffold modulates monocyte to promote cell homing, angiogenesis and bone regeneration. Biomaterials.

[140] Aimé C., Mosser G., Pembouong G., Bouteiller L., Coradin T. (2012). Controlling the nano–bio interface to build collagen–silica self-assembled networks. Nanoscale.

[141] Kahn J.L., Eren N.M., Campanella O., Voytik-Harbin S.L., Rickus J.L. (2016). Collagen-fibril matrix properties modulate the kinetics of silica polycondensation to template and direct biomineralization. J. Mater. Res..

[142] Rickus J.L., Harbin S.L., Kahn J.L. (2016). Cell-Collagen-Silica Composites and Methods of Making and Using the Same. Patent.

